# Structural, Physical, and Antifungal Characterization of Starch Edible Films Added with Nanocomposites and Mexican Oregano (*Lippia berlandieri* Schauer) Essential Oil

**DOI:** 10.3390/molecules24122340

**Published:** 2019-06-25

**Authors:** Rocio Aguilar-Sánchez, Ricardo Munguía-Pérez, Fatima Reyes-Jurado, Addí Rhode Navarro-Cruz, Teresa Soledad Cid-Pérez, Paola Hernández-Carranza, Silvia del Carmen Beristain-Bauza, Carlos Enrique Ochoa-Velasco, Raúl Avila-Sosa

**Affiliations:** 1 Facultad de Ciencias Químicas, Benemérita Universidad Autónoma de Puebla. Puebla, Puebla 72420, Mexico; rocio.aguilar@correo.buap.mx (R.A.-S.); fatima.reyes.jurado@iberopuebla.mx (F.R.-J.); addi.navarro@correo.buap.mx (A.R.N.-C.); teresolcid@gmail.com (T.S.C.-P.); phernandezcarranza@hotmail.com (P.H.-C.); silvia.beristain@udlap.mx (S.d.C.B.-B.); carlosenriqueov@hotmail.com (C.E.O.-V.); 2 Laboratorio de Micología, Centro de Investigaciones en Ciencias Microbiológicas, Instituto de Ciencias, Benemérita Universidad Autónoma de Puebla, Puebla 72420, Mexico; ricardo.munguia@correo.buap.mx; 3 Departamento de Ciencias de la Salud, Universidad Iberoamericana Puebla, San Andrés Cholula, Puebla 72820, Mexico

**Keywords:** gas permeability, thickness, porosity, bentonite, halloysite, *Fusarium* spp., *Aspergillus niger*

## Abstract

The aim of this study was to evaluate the structural, physical, and antifungal characteristics of starch edible films added with nanocomposites and Mexican oregano (*Lippia berlandieri* Schauer) essential oil (EO). Starch edible films were formulated with Mexican oregano EO (0%, 1%, or 2% *v*/*v*) and bentonite or halloysite (2%). Physical properties such as L* (luminosity), hue, film thickness, and O_2_ and CO_2_ permeability were determined. Structural analysis was carried out via atomic force microscopy (AFM). Antifungal activity against *Aspergillus niger, Fusarium* spp., and *Rhizopus* spp. was evaluated. The addition of EO and nanocomposites reduced luminosity, providing color to the edible films. Film thickness increased through the addition of EO concentration. O_2_ and CO_2_ permeability was increased by bentonite/EO films, and for halloysite films, CO_2_ permeability decreased as EO concentration increased. The addition of EO with both nanocomposites shows an evident morphological change in film structure, decreasing pore density and increasing pore size. In general, Mexican oregano EO added to edible starch films has an adequate fungicidal effect. The most sensitive microorganism tested was *A. niger.* Edible films added with Mexican oregano EO and nanocomposites show better physical and antifungal properties due to an adequate structural change in the biopolymer matrix.

## 1. Introduction

Starch is one of the most abundant natural biopolymers and is particularly attractive due to its low-cost biodegradability, edibility, ease of chemical modification and sustainability, which is why many studies have focused on its application to designing edible films and coatings [1,2]. Starch edible films have many advantages over traditional food packaging such as plastics, which have low degradability and are even toxic to humans. Moreover, in recent years, starch edible films have been able to incorporate antimicrobial agents to provide microbiological stability to foods because they serve as carriers of many compounds, which include naturally occurring antimicrobials, such as essential oils (EOs) [3,4]. 

EOs’ incorporation into edible films to obtain active food packaging is of key interest for food preservation [5]. These compounds seem to be promising additives which could interact with film-forming polymers and improve edible films’ physical and functional properties via the production of cross-links between polysaccharides [6], which can extend product shelf life and reduce the risk of pathogen growth on food surfaces [7]. In this aspect, some extracts have evaluated, for example, extracts of rosemary, peppermint oil, thyme, olive, and ginger and found that they showed excellent antimicrobial potential, and their incorporation in films considerably delayed the microbial growth [8]. Mexican oregano (*Lippia berlandieri* Schauer) EO has the potential to be applied in starch edible films to inhibit *Aspergillus niger* and *Penicillium* spp. growth at low concentrations, having a great impact on their lag phase. Therefore, it may be an alternative to formulate effective antifungal edible films [3]. However, starch edible films have not been widely used in the packaging industry to replace the conventional petroleum plastic, mainly because of their poor mechanical, barrier, and technological properties [2,9,10]. To overcome these limitations, nanocomposites are a new concept of materials which are produced using reinforcement particles with at least one dimension at the nanoscale; these compounds may be incorporated into biopolymer films. Nanocomposites exhibit mechanical, thermal, optical, physiochemical, and barrier properties that are far better than those of pure polymers [11,12,13].

An edible film added with nanocomposites and antimicrobials is particularly desirable because of its integrity and acceptable structural barrier properties imparted by the nanocomposite matrix. The incorporation of EO to edible films requires smaller concentrations compared to direct EO application to inhibit different types of microorganisms [3]. In order to take full advantage of these benefits, it is necessary that the nanoparticles are uniformly dispersed in the polymeric matrix once the film-forming solution is made and subsequently added to the antimicrobial substance in question [14,15]. Although there are a series of investigations in the characterization of the physical and mechanical properties of this type of film, there is little evidence of their antimicrobial effect on certain films with cellulose nanocomposites, especially in pathogenic bacteria such as *Staphylococcus aureus*, *Escherichia coli*, *Listeria monocytogenes*, and *Salmonella typhimurium* [16,17]. The application of food-grade nanocomposites in edible films has a promising future because it has been found to have a positive impact on mechanical properties, with increasing film strength and improving barrier properties. Further, nanoparticles can be used as carriers of antimicrobial agents and additives [18].

Therefore, the aim of this study was to evaluate the structural, physical, and antifungal characteristics of starch edible films added with nanocomposites and Mexican oregano EO.

## 2. Results and Discussion

### 2.1. EO Chemical Composition

**Figure 1:**
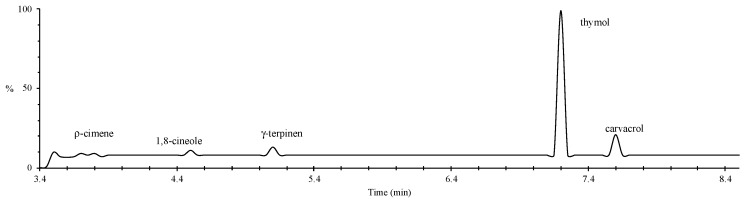
Mass spectra of Mexican oregano (Lippia berlandieri Schauer) essential oil.

Mass spectra of Mexican oregano EO are presented in Figure 1. Several compounds are related to its chemical composition: the isomeric monoterpenes thymol (2.103 g/mL) and carvacrol (0.533 g/mL) and their corresponding biosynthetic precursors, detected in very low concentrations; p-cymene; 1,8-cineole; and γ-terpinen. The obtained concentrations agree with previous reports [19,20]. The main component of Mexican oregano EO is thymol, and its concentrations are greater when the extracts are obtained from younger plants [21].

### 2.2. Physical Properties

Table 1 shows the physical properties of edible films formulated with nanocomposites and Mexican oregano EO. A higher value of luminosity was obtained for control edible coating; however, once EO is added, luminosity decreases significantly (*p* < 0.05) for bentonite films, making them opaquer. However, halloysite films’ luminosity values showed no significant differences with control films (*p* > 0.05). When nanocomposites and EO were added, the luminosity value was altered. In the case of bentonite films, it was lower than in halloysite films; this may be due to the nanocomposites structure itself, due to the fact that the bentonite structure was type 2:1 and the halloysite structure was type 1:1: Since the structure was less compact, films with halloysite give way to light and have a higher luminosity value [22]. Moreover, Flaker et al. [23] confirmed that the addition of nanocomposites to edible films reduces luminosity due to the smoothness of its surfaces, which influences light diffraction.

Hue value differences in each of the films analyzed can be observed due to the type of nanocomposite and Mexican oregano EO concentration. A comparison was made between the films, finding significant differences (*p* < 0.05), which indicates that all films are optically different in relation to the color. The films acquired the proper color of the EO and the different nanocomposites, especially with the halloysite, which has a characteristic white color, and the bentonite, which is brown, giving different tonalities for each of the analyzed films. Mexican oregano EO color intensified due to their concentration. In general, depending on the number of nanoparticles added, the films exhibited changes in brightness and different color values, in accordance with the studies carried out by Voon et al. [24].

For thickness values, the control film was the thinner one, and this was affected by adding EO due to different concentrations in the polymer matrices. The thicknesses were increased when adding the nanocomposites and EO significantly (*p* < 0.05). This effect can be partially related to film solution density due to a larger molecular contact between starch CH groups and Mexican oregano EO components, weakening the polymer chain aggregation forces and producing a more open matrix, leading to a higher film thickness [25].

In gas permeability, for both types of nanocomposites, there is a significant increase (*p* < 0.05) in oxygen permeability with respect to the control film. For CO_2_ permeability, there are different behaviors. For example, in bentonite edible films, as the EO concentration increases, the permeability significantly decreases (*p* < 0.05) compared to control films. On the other hand, for halloysite films without EO, the permeability value is higher than in the control and decreases as EO concentration increases. The combination of starch with nanocomposites and Mexican oregano EO contributes to a modification on films structures. The disruption of hydrogen bonds may create additional sites for the dissolution of these gases and increase the mobility of molecules through film structure. According to Slavutsky et al. [26], the combination of nanocomposites and EO decreases gas permeability due to a decrease in hydrophilicity of the film and the tortuous pathway formed by the nanocomposites incorporation even though that CO_2_ has the lowest kinetic diameter. Moreover, CO_2_ strongly interacts with polar groups present in the film.

### 2.3. Structural Analyses

**Figure 2:**
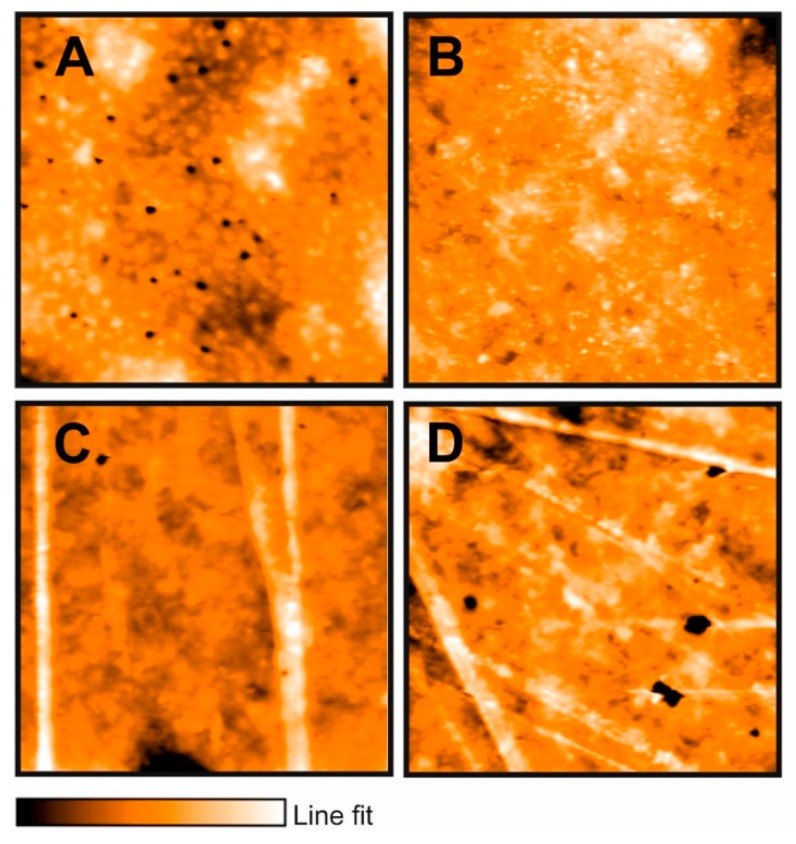
Representative atomic force microscopy (AFM) images of (**A**) starch (**B**) starch/bentonite, (**C**) starch/bentonite/Mexican oregano (*Lippia berlandieri* Schauer) essential oil 1%, and (**D**) starch/bentonite/Mexican oregano (*Lippia berlandieri* Schauer) essential oil 2%. Conditions: Static force, set point 55 nN. Area: 50 × 50 μm. Line fit (color intensity) shows the vertical profile of the sample. The lightest region is the highest point, and the dark region corresponds to depressions in the material: (**A**) 0.26 μm, (**B**) 0.17 μm, (**C**) 0.464 μm, and (**D**) 0.3 μm.

**Figure 3:**
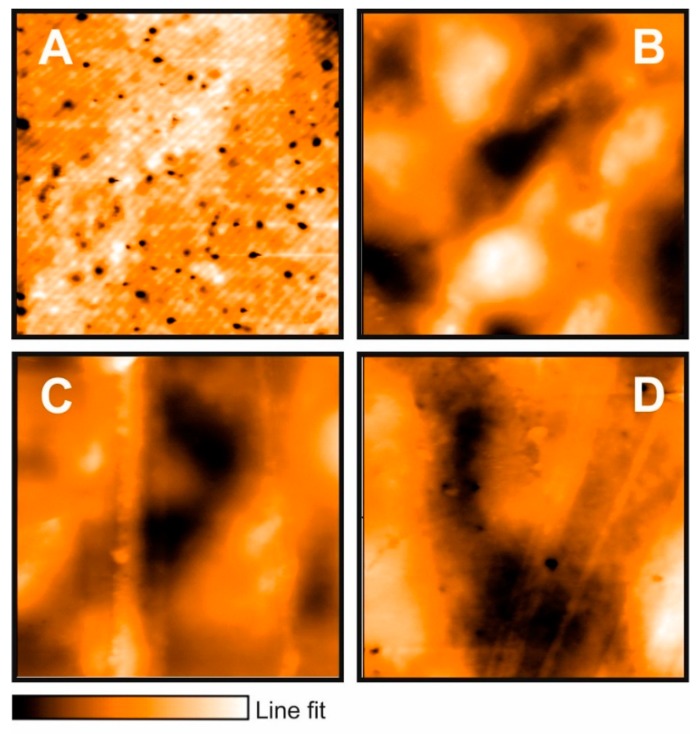
Representative AFM micrographs of (**A**), starch (**B**), starch/halloysite, (**C**) starch/halloysite/Mexican oregano (*Lippia berlandieri* Schauer) essential oil 1%, and (**D**) starch/halloysite/Mexican oregano (*Lippia berlandieri* Schauer) essential oil 2%. Conditions: Static force, set point 55 nN. Area: 50 × 50 μm. Line fit (color intensity): Line fit (**A**) 0.2 μm, (**B**) 0.96 μm, (**C**) 1.35 μm, (**D**) 1.37 μm.

Representative low-resolution atomic force microscopy (AFM) images of starch and starch–nanocomposite films are shown in Figure 2 and Figure 3. In Figure 2A and Figure 3A, the microstructure of starch (control) film is presented. A uniform film can be observed with a high density of deep depressions that are evenly distributed in the entire film. These features were found to be pores of approximately 0.15 ± 0.01 μm depth and 0.9 ± 0.2 μm diameter, as estimated from the height profiles. Porosity is essential to define practical applications and permeability for the films.

The morphology for the hybrid microstructure of bentonite powder modified starch film (Figure 2B) showed a more compact surface structure. It was found that the density of depressions (pore density) decreased considerably (from 0.66/mm^2^ to 0.07/mm^2^), indicating that barrier properties against water vapor, moisture and gases, and mechanical properties of starch composite films can be reinforced through the addition of bentonite. Further, the rugosity slightly decreased; the obtained surface roughness average for the bentonite–starch film was 21.4 ± 7 nm, while for the control film, it was 33.4 ± 10 nm. Roughness is one of the most important surface properties, since it is involved in flow capabilities, membrane hydrophilicity, and contact angle [27].

When halloysite was combined with starch (Figure 3B), the morphology showed an evident structural change related to the formation of non-uniform agglomerates formed along the composite film, with an inhomogeneous distribution of halloysite particles within the polymer matrix. Compared to the control film, the rugosity increased twice, and the pores’ density decreased (form 0.66/mm^2^ to 0.02 mm^2^). Based on the line fit (color intensity bar) at the bottom of Figure 3, it can be observed that the thickness rises for the halloysite–starch hybrid as a consequence of the agglomerate formation.

After addition of oregano EO to the composite films, starch/bentonite or starch/halloysite, the structure changed, showing a fibrous-like structure which can be expected as a result of the interactions of composite films with oregano EO compounds, such as thymol and carvacrol, since for composite films prepared with these standard substances, the structure is also fibrous-like (results not shown). The highest roughness values were obtained for the composite films added with 2% of EO, which could highly influence local mass transfer, contact angle and film hydrophilicity/hydrophobicity after the addition of nanocompounds.

This surface texture change may be due to the formation of agglomerates caused by the interactions between all the components in the film-forming solution, which persisted in films after the drying process [23]. Film roughness can aid in keeping smaller particles such as bacteria or spores on films surfaces; in this way, EO can exert their effect when released from the formed structures. The presence of nanocomposites in films improves the structural properties [26], and therefore, gas transport properties and materials can be modulated through them [28]. According to Sales Monteiro et al. [29], bentonite and halloysite are used as filling materials in starch matrix due to its structure: Starch is highly hydrophilic and therefore has a low affinity with nanocomposites, with an incomplete interaction with biopolymer. Moreover, when EO is added, the presence of different functional groups of all their compounds provides an increase of the affinity between nanocomposites and starch, resulting in an exfoliated surface.

### 2.4. Antifungal Properties

**Figure 4:**
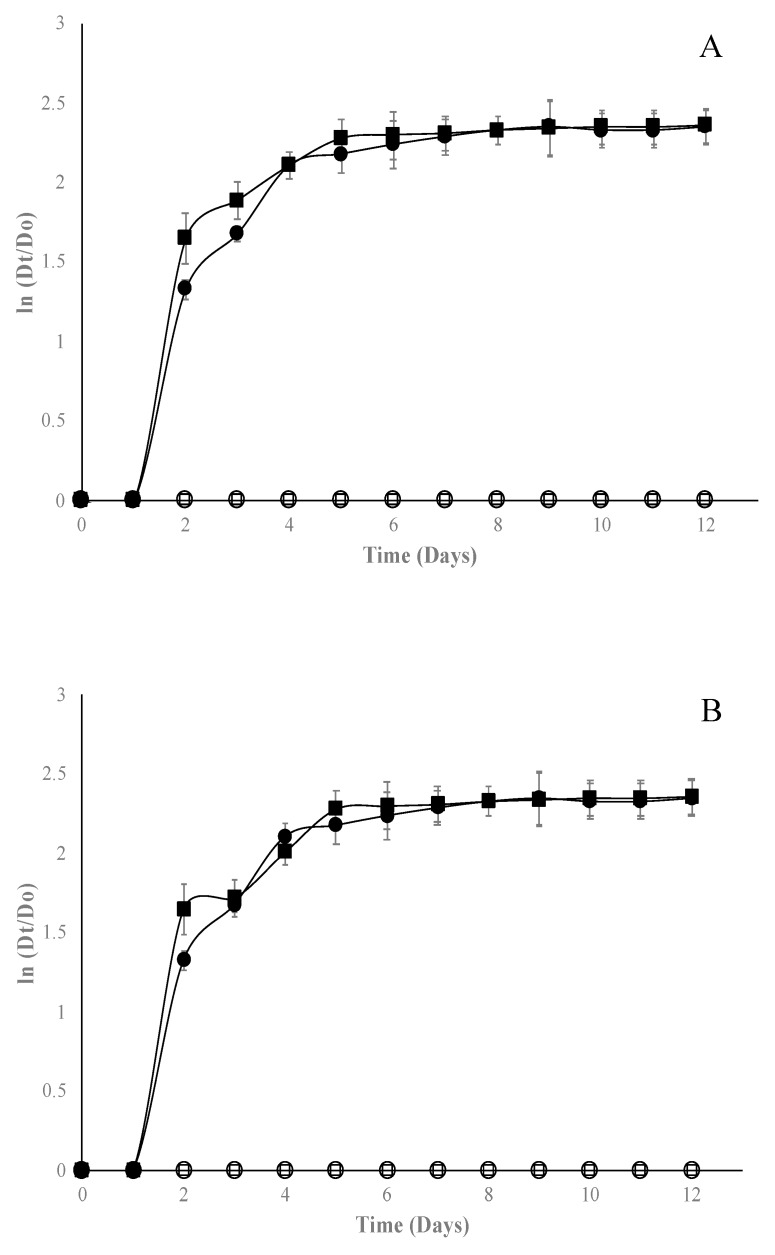
Effect of starch edible films added with selected concentrations of Mexican oregano (*Lippia berlandieri* Schauer) essential oil (control [■], 0% [●], 1% [☐], and 2% [⭘]) on *Aspergillus* *niger* growth with different nanocomposites, bentonite (**A**) or halloysite (**B**).

**Figure 5:**
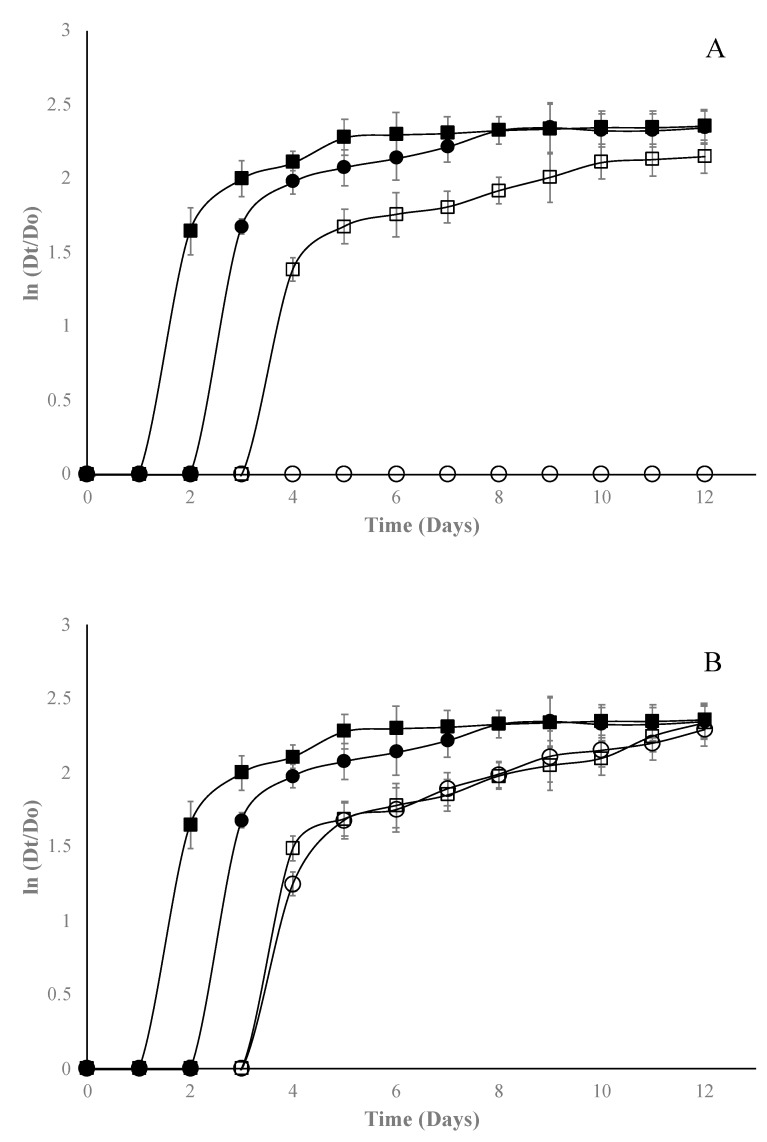
Effect of starch edible films added with selected concentrations of Mexican oregano (*Lippia berlandieri* Schauer) essential oil (control [■], 0% [●], 1% [☐], and 2% [⭘]) on *Fusarium* spp. growth with different nanocomposites, bentonite (**A**) or halloysite (**B**).

In general, the EO of Mexican oregano added to edible starch films has an adequate fungicidal effect. The most sensitive microorganism was *A. niger* (Figure 4), which was inhibited with concentrations of 1% for both types of film. Conversely, *Fusarium* spp. was the most resistant microorganism (Figure 5); it could only be inhibited with films with bentonite and EO at 2%.

**Figure 6:**
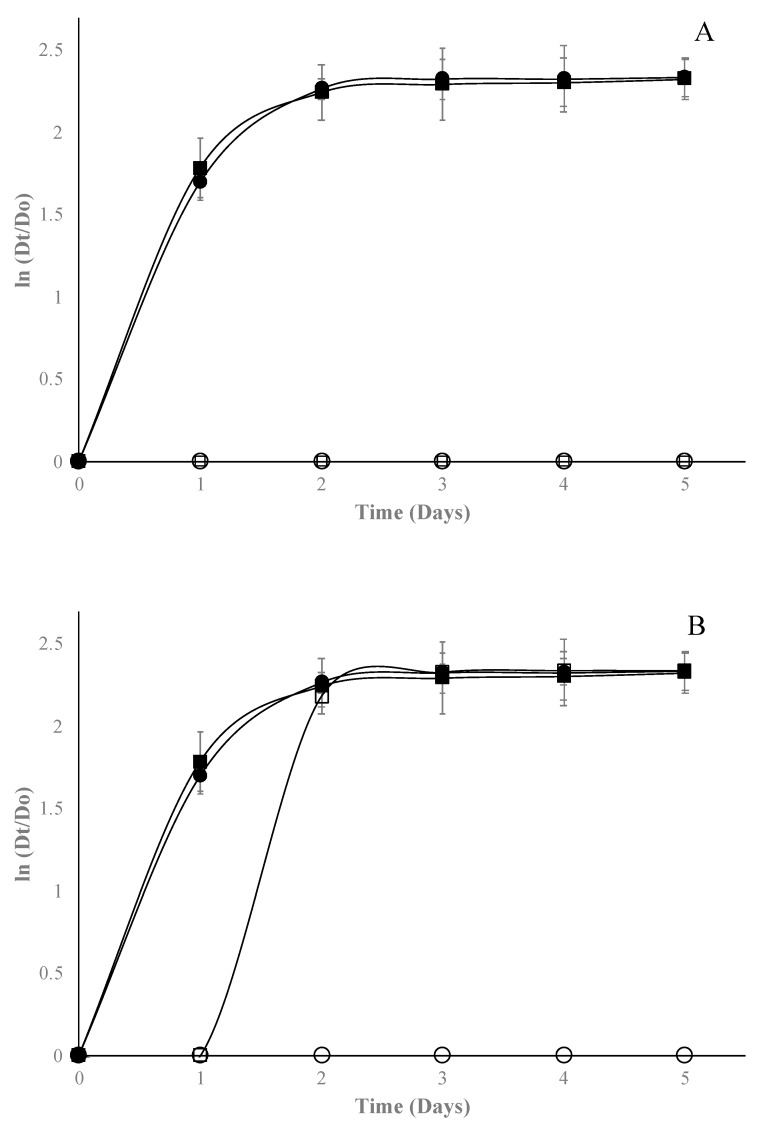
Effect of starch edible films added with selected concentrations of Mexican oregano (*Lippia berlandieri* Schauer) essential oil (control [■], 0% [●], 1% [☐], and 2% [⭘]) on *Rhizopus* spp. growth with different nanocomposites, bentonite (**A**) or halloysite (**B**).

When Gompertz parameters were analyzed (Table 2), it can be observed for *Fusarium* spp. that there is only a fungistatic effect, prolonging on average two days more the lag phase, which was significantly different (*p* < 0.05) to control films. Finally, for *Rhizopus* spp. (Figure 6), bentonite films were more effective than halloysite films, since they could be inhibited from 1% of EO concentration; the λ parameter shows a fungistatic effect. For the three fungal genera, as the EO concentration increases, λ is affected, which increases significantly (*p* < 0.05) with respect to control edible films. Since no studies on the use of nanocomposites and Mexican oregano EO are reported in the available literature, it is impossible to compare the results obtained in this study; however, a few studies were reported on the antifungal effect of edible films added with other EO oil. For example, the authors of [30,31] managed to inhibit the growth of *A. niger* with gelatin-based nanocomposite films containing nanochitin as an antimicrobial in concentrations near 5%. Ortega-Toro et al. [32] added *Aloe vera* to edible starch films to inhibit *Fusarium oxysporum* at concentrations higher than 2%. Acosta et al. [4] incorporated oregano EO into edible starch–gelatin blended films, finding inhibition at concentrations over 2% for *Fusarium oxysporum.* Finally, Escamilla-García et al. [25] added different EOs (anise, orange, and cinnamon) to edible chitosan–zein films to inhibit *Rhizopus* spp. in concentrations higher than 4%. Thus, the use of nanocomposite and Mexican oregano EO showed better results than those obtained by other researchers. Portillo-Ruiz and et al. [33] reported antifungal activity in vitro of Mexican oregano EO on *A. niger* and *Penicillium* spp. but observed only a fungistatic effect at concentrations near 4% of EO. Incorporation of EOs to edible films requires smaller concentrations compared to direct application to inhibit different types of microorganisms [34,35].

## 3. Materials and Methods

### 3.1. Reagents, Culture Media and EO

All chemical reagents used in this study were obtained from Sigma-Aldrich, Inc. (Toluca, Mexico), culture media from Bioxon (Mexico City, Mexico), and Mexican oregano (*Lippia berlandieri* Schauer) EO was obtained and processed at CIReNa (Natural Resources Research Center, DGTA, Salaices, Chihuahua).

### 3.2. Film Preparation

Edible films were made by the casting method [36], which consists of drying the corresponding film forming solution (FFS) that has been applied on a support. One gram of high amylose corn starch was mixed with 10 mL of previously sterilized 0.25 N sodium hydroxide and 10 mL of distilled water. FFS was maintained for 60 min under stirring conditions. Starch FFS was gelatinized in a shaker water bath at 78–80 °C for 10 min; when the solution was decreased to 40 °C, glycerol (1.2% *v*/*v*) was added. FFSs were mixed (IKA High Performance Disperser T18, Chicago, IL, USA) under aseptic conditions at 20,000 rpm for 1 min at room temperature with the incorporation of Mexican oregano EO at 0%, 1%, or 2% (*v*/*v*). Then, nanocomposites (bentonite or halloysite) were added (2% *w*/*v*) to obtain a final concentration and poured into 60 mm inner diameter sterile Petri dishes. Films were prepared with 7 mL of FFS per Petri dish (1 film), dried under 0.35 kg/cm^2^ vacuum at 30 °C for 12 h. Films were kept in sealed Petri dishes at 4 °C until analysis.

### 3.3. Chemical Characterization of Mexican Oregano Essential Oil

The EO was analyzed with a Perkin Elmer Turbo Mass Gold MS-Auto system XLTM gas chromatography/mass spectrometry (GC/MS) system (Perkin-Elmer, Norwalk, CT, USA). An Altech capillary column (30 m, 0.32 mm i.d., and 0.1 mm film thickness) was used for separation of components. Helium as carrier gas had a flow rate of 26 cm/s. The injector temperature was maintained at 220 °C. A temperature program with a total run time of 25 min was used. The column temperature, after an initial isothermal period of 1 min at 55 °C, was increased to 120 °C at a rate of 10 °C/min and maintained at this temperature for 1 min. Then, the column temperature was further increased to 220 °C at a ramp rate of 20 °C/min and maintained at this temperature for 3.42 min. Mass spectrum conditions had an ionization energy of 70 eV and ion source temperature of 250 °C in single ion monitor mode. The obtained spectra were compared with respective mass spectra of pure compounds, and also with the mass profile of the same compounds available from the National Institute of Standard Technology (NIST) library, USA.

### 3.4. Physical Films Properties

#### Color

L* (luminosity), a* (+ red, − green), and b* (+ yellow, − blue) color parameters of CIELab scale were evaluated using a precise colorimeter reader (TCR 200, TIME High Technology, Beijing, China). Color parameters were evaluated in three different points on edible film surface. Hue was calculated following the next equation:

(1) $$Hue=tan^{-1}\left(\frac{b^{*}}{a^{*}}\right),$$

where L*, a* and b* are the edible films color parameters [37].

#### Thickness

Film thickness (μm) was determined on every film formulation averaging measurements at 5 points of each film using a micrometer (Scala, 111-B, Mexico City, Mexico).

### 3.5. Films O_2_ and CO_2_ Permeability

Oxygen and carbon dioxide permeability measurements were carried out according to the isostatic method proposed by Mujica-Paz and Gontard [38] with some modifications. Edible film was placed in a permeation cell which consisted of two stainless steel chambers separated by a film sample. Gas testing was injected into one chamber, and helium was directed into the other side. Chamber pressures were equalized and maintained at atmospheric pressure. The gas was left to stand for 100 min, and then a sample was taken from the gas chamber using a syringe adapted with a three-way stopcock to be analyzed using a gas analyzer (Buck Scientific, 910, East Norwalk, CT, USA), equipped with a Flame Ionization Diode (FID) detector. At steady state (constant oxygen or carbon dioxide concentration in the helium stream), flow rate and helium gas composition stream were measured and used to calculate the oxygen or carbon dioxide permeability. Permeability (P) was calculated following Equation (2):

(2) $$P=\frac{J\left(∆x\right)}{A\left(∆p\right)},$$

where *P* is the CO_2_ and O_2_ permeability (mL of gas/m*s*bar), *J* is the transmission rate of CO_2_ or O_2_ (mL/s), ∆*x* is the film thickness (m), *A* is the film surface (m^2^), and ∆*p* is the differential partial pressure of the permeant gas across the film (bar).

### 3.6. Films Structural Analysis

Structural analysis was carried out via AFM, employing a Nanosurf Naio-AFM microscope (Nanosurf, Liestal, Switzerland) provided with silicon carbide tips, and the measurements were done in contact mode. The acquisition and analysis data were performed with the Naio Control Software Version 3.1. All measurements were done in air, under static force operating mode with a set point of 55 nN. Structural analysis was performed with five different samples and four regions of each sample. The shown images are representative of each sample. The evaluated parameters were pore size, pore density, and pore deepness, as well as roughness. The data are representative of 1000 mm^2^ in total and are reported in mm^2^. Pore size and surface roughness were estimated by performing an analysis of at least four samples.

### 3.7. Antifungal Effect

*Aspergillus niger, Fusarium* spp., and *Rhizopus* spp. were obtained from Laboratorio de Micología of Instituto de Ciencias, Benemérita Universidad Autónoma de Puebla, Puebla, Mexico. They were cultivated for 5 days at 25 °C on potato–dextrose agar plates acidified with 10% of tartaric acid. Dishes containing 5-d cultures were used to recover fungal spores, which were obtained by pouring 9 mL of sterile physiological water on the agar plate surface, followed by a gentle scraping using a sterile rake to remove the maximum quantity of spores. After that, spore suspensions were transferred into sterile tubes. The number of spores present in the suspension was determined using a hemocytometer and an optical microscope (Zeiss Primo Star, Göttingen, Germany) and expressed as the number of spores per milliliter (spores/mL). Suspensions were serially diluted to approximately 1 × 10^6^ spores/mL [39] and then 10 μL of spore suspension on sterile PDA agar. Dishes were dried in a flow hood (Labconco, Kansas City, MO, USA) at room temperature for 30 min, and then edible films were placed over each inoculated place and incubated at 25 °C. A growth control was prepared in parallel, to ensure that viable organisms were present. Radial growth was measured every 24 h during 12 days. Each test was performed by triplicate.

### 3.8. Modeling andStatistical Analysis

Growth curves were generated from experimental data by plotting ln (*D_t_*/*D_o_*), where *D_t_* is the average colony diameter at time *t* (d) and *D_o_* (cm) is the average colony diameter at the initial time. Data were fitted using the modified Gompertz model [40], from which biological parameters were estimated by nonlinear regression; in order to validate the results, the coefficient of determination (*R*^2^_adj_) was determined to each growth curve using KaleidaGraph 3.51 (Synergy Software, Reading, PA, USA):

(3) $$\ln\left(\frac{D_{t}}{D_{o}}\right)=A*exp\left\{-exp\left[\left(\frac{\upsilon_{max}*e}{A}\right)\left(\lambda -t\right)+1\right]\right\},$$

where *A* is the maximum mold growth achieved during the stationary phase, *υ*_max_ is the maximum specific growth rate (cm/d), λ is the lag time (d), and *e* = 2.7182.

All parameters were analyzed using analysis of variance (ANOVA) with Minitab 17 software (Minitab Inc., State College, PA, USA). A *p*-value of 0.05 was used to decide significant differences among averages (Tukey’s test).

## Figures and Tables

**Table 1:** Luminosity (L*), hue (h*), film thickness, and O_2_ and CO_2_ film permeability (mean ± standard deviation) values of starch edible films added with selected concentrations of Mexican oregano (*Lippia berlandieri* Schauer) essential oil and nanocomposites (bentonite or halloysite). Mean values in the same column not sharing a common superscript are significantly different (*p* < 0.05).

		L*	h*	Thickness (μm)	O_2_ Permeability (mL O_2_/m × s × bar)	CO_2_ Permeability (mL CO_2_/m × s × bar)
	Control	58.34 ± 1.12 ^a^	0.82 ± 0.14 ^a^	10.24 ± 2.15 ^a^	1.47 × 10^−6^ ± 3.32 × 10^−8 a^	3.14 × 10^−8^ ± 7.55 × 10^−10 a^
Bentonite	0%	51.45 ± 1.44 ^b^	0.61 ± 0.08 ^b^	13.32 ± 2.31 ^a^	2.34 × 10^−6^ ± 3.72 × 10^−8 b^	3.30 × 10^−8^ ± 5.32 × 10^−10 a^
	1%	52.31 ± 1.31 ^b^	0.34 ± 0.05 ^c^	13.46 ± 1.96 ^a^	2.20 × 10^−6^ ± 5.53 × 10^−8 b^	2.11 × 10^−8^ ± 5.02 × 10^−10 b^
	2%	50.67 ± 0.99 ^b^	0.15 ± 0.04 ^d^	16.34 ± 0.92 ^b^	2.12 × 10^−6^ ± 3.09 × 10^−8 b^	1.71 × 10^−8^ ± 0.21 × 10^−10 c^
Halloysite	0%	57.73 ± 2.03 ^a^	0.75 ± 0.13 ^a^	8.34 ± 1.16 ^a^	1.98 × 10^−6^ ± 5.28 × 10^−8 c^	3.53 × 10^−8^ ± 0.18 × 10^−10 d^
	1%	59.44 ± 1.72 ^a^	0.79 ± 0.11 ^a^	17.23 ± 1.52 ^b^	1.64 × 10^−6^ ± 1.80 × 10^−8 d^	2.91 × 10^−8^ ± 0.28 × 10^−10 e^
	2%	57.59 ± 1.47 ^a^	1.14 ± 0.18 ^e^	18.21 ± 1.64 ^b^	1.71 × 10^−6^ ± 1.53 × 10^−8 d^	2.26 × 10^−8^ ± 0.44 × 10^−10 f^

**Table 2:** Modified Gompertz model parameters^+^ (mean ± standard deviation) for *A. niger*, *Fusarium* spp., and *Rhizopus* spp. growth curves of starch edible films added with selected concentrations of Mexican oregano (*Lippia berlandieri* Schauer) essential oil and nanocomposites (bentonite or halloysite). Mean values for each microorganism in the same column not sharing a common superscript are significantly different (*p* < 0.05).

		A	ν_max_ (1/day)	λ (day)
*A. niger*				
	Control	2.45 ± 0.72 ^a^	0.65 ± 0.14 ^a^	1.05 ± 0.44 ^a^
Bentonite	0%	2.51 ± 0.31 ^a^	0.53 ± 0.11 ^a^	1.23 ± 0.67 ^a^
	1%	-	-	>12 ^b^
	2%	-	-	>12 ^b^
Halloysite	0%	2.36 ± 0.16 ^a^	0.58 ± 0.08 ^a^	1.21 ± 0.22 ^a^
	1%	-	-	>12 ^b^
	2%	-	-	>12 ^b^
*Fusarium* spp.				
	Control	2.31 ± 0.52 ^a^	0.45 ± 0.09 ^a^	1.32 ± 0.34 ^a^
Bentonite	0%	2.35 ± 0.12 ^a^	0.55 ± 0.10 ^a^	2.09 ± 0.25 ^b^
	1%	2.16 ± 0.21 ^a^	0.60 ± 0.06 ^a^	3.15 ± 0.32 ^c^
	2%	-	-	>12 ^d^
Halloysite	0%	2.31 ± 0.23 ^a^	0.65 ± 0.08 ^a^	2.13 ± 0.17 ^b^
	1%	2.23 ± 0.16 ^a^	0.57 ± 0.16 ^a^	3.09 ± 0.14 ^ac^
	2%	2.19 ± 0.25 ^a^	0.58 ± 0.11 ^a^	3.14 ± 0.07 ^c^
*Rhizopus* spp.				
	Control	2.33 ± 0.17 ^a^	0.76 ± 0.19 ^a^	0.12 ± 0.02 ^a^
Bentonite	0%	2.28 ± 0.08 ^a^	0.67 ± 0.21 ^a^	0.08 ± 0.01 ^a^
	1%	-	-	>12 ^b^
	2%	-	-	>12 ^b^
Halloysite	0%	2.31 ± 0.11 ^a^	0.51 ± 0.14 ^a^	0.13 ± 0.04 ^a^
	1%	2.28 ± 0.09 ^a^	0.56 ± 0.12 ^a^	1.12 ± 0.32 ^b^
	2%	-	-	>12 ^c^

A: maximum fungal growth in the stationary phase; ν_max_: maximum specific growth rate; λ: lag phase. -: no growth.
